# Health Teaching for the Young

**Published:** 1920-01-10

**Authors:** 


### Health Teaching for the Young.

One of the most interesting developments in the direction of social reconstruction in the near future will be the formation of the new continuation classes for young people who have left school and are at work. The opportunities and scope in this work are enormous, and the public must rouse itself and see that the opportunities are not wasted. One of the ways in which great use may be made of the classes is in the direction of health propaganda. The young people will be of an age when health guidance is very necessary, and if this is given judiciously and sympathetically, preferably by qualified doctors, they will readily respond and show interest. Some health talks were given to young people in connection with the classes for unemployed juveniles on the cessation of hostilities and were much appreciated. The pupils will be in work at factories and shops, and their outlook on life on the threshold of their career is naturally one of independence and emancipation from the restraining influences of school and, perhaps, home. This factor must of necessity be taken into account and the attitude of the teacher adapted accordingly, or much harm will be done, especially if real good is to be done in the region of education in health matters. The young people must be made to feel the urgent need of the education and to regard it as a demand created by themselves. In the schools they have had medical inspection and treatment, and the good effects of this work should be carried forward into the continuation school.

